# Human MxA is a potent interspecies barrier for the novel bat-derived influenza A-like virus H18N11

**DOI:** 10.1080/22221751.2019.1599301

**Published:** 2019-04-04

**Authors:** Kevin Ciminski, Johanna Pulvermüller, Julia Adam, Martin Schwemmle

**Affiliations:** a Institute of Virology, Medical Center – University of Freiburg, Freiburg, Germany; b Faculty of Medicine, University of Freiburg, Freiburg, Germany

**Keywords:** Bat influenza A virus, H18N11, chimeric influenza A virus, PR8-H18N11, MxA, interspecies barrier, zoonotic spill-over

## Abstract

The human innate immune factor MxA represents an effective interspecies barrier for zoonotic influenza A viruses (IAVs) of animal origin. Accordingly, human but not avian IAVs efficiently escape the antiviral activity of MxA due to adaptive mutations in their viral nucleoprotein. Partial MxA resistance can be acquired in intermediate hosts such as swine, which possess an antivirally active Mx1 protein. Intriguingly, Mx1 of the bat *Carollia perspicillata*, a host of the recently discovered bat influenza A-like virus H18N11, is antivirally active against avian IAVs, thus raising the question whether H18N11 has undergone a preadaptation to human MxA. Here, by utilizing a chimeric bat influenza virus, PR8-H18N11, we demonstrate that MxA efficiently blocks viral replication *in vitro* as well as in MxA transgenic mice. Nevertheless, the H18N11 nucleoprotein exhibits partial MxA resistance in a polymerase reconstitution assay*,* suggesting that a certain degree of MxA preadaptation occurred. Together, our data indicate a currently reduced risk for H18N11 to overcome the human restriction factor MxA. Further adaptive mutations in NP are required to facilitate full MxA escape.

## Introduction

Zoonotic infections with influenza A viruses (IAVs) pose a constant threat to the human population, where they can cause severe disease in individuals and potentially also give rise to global pandemics [1–5]. In humans, the type I and III interferon (IFN)-induced large dynamin-like GTPase Mx1 (in humans commonly known as MxA) represents a potent interspecies barrier that restricts such spill-over infections. Despite their close phylogenetic relationship, all known avian Mx1 proteins do not exhibit any antiviral activity against IAVs [6,7]. Hence, in order to cross the human species barrier, avian IAVs have to acquire certain adaptive mutations in their viral nucleoprotein (NP) to overcome MxA restriction [8–10]. Indeed, while these MxA escape amino acid patches in NP are highly conserved in all circulating human isolates, they are virtually absent in avian IAVs, suggesting that human-adapted IAVs are under constant evolutionary pressure by MxA restriction [8,11]. Originally, these Mx resistance mutations were identified by mutational approaches to be located in distinct patches in the NP body domain of the human-adapted 1918 and 2009 pandemic IAV strains [8]. Substitution of specific key residues in NP of Mx sensitive avian with those of human IAVs resulted in increased Mx resistance *in vitro* and *in vivo* [8,10,12]. Importantly, as demonstrated for the 2009 pandemic H1N1 virus and more recently for a Eurasian avian-like swine IAV, MxA escape mutations in NP, and thus a partial MxA resistance, can be acquired in intermediate hosts like swine that express an antivirally active Mx1 protein [8,13].

In 2012 and 2013 two genome sequences of influenza A-like viruses, designated H17N10 and H18N11, were identified in New World bat specimens [14,15]. This discovery challenged the hitherto known IAV host range and raised concerns about potential spill-over of these novel bat-derived influenza viruses to humans. Intriguingly, bat Mx1 (bMx1) from *Carollia perspicillata* (*C. perspicillata*), a host of H18N11 [15], exerts antiviral activity against avian IAVs similar to the human orthologue MxA in a polymerase reconstitution assay [16], implying that bats, similar to swine could serve as an intermediate host, in which MxA preadaptation occurs. In order to evaluate whether the ongoing circulation of H18N11 in presence of antivirally active bMx1 resulted in partial MxA escape, we generated a bat chimeric virus PR8-H18N11, expressing the A/Puerto Rico/8/1934 (PR8) surface glycoproteins plus the six internal gene segments of H18N11 and performed infection studies in MxA-expressing cells and mice, carrying the human *MX1* gene locus as a transgene [12].

## Material and methods

### Cells

MDCKII (Merck; #00062107) and HEK293 T cells (ATTC; CRL-3216) as well as MDCK-SIAT1, MDCK-SIAT1-MxA, MDCK-SIAT1-MxA_T103A_ that were kindly provided by Jesse D. Bloom (Fred Hutchinson Cancer Research Center, United States [9]) were cultured in Dulbecco’s modified Eagle’s medium (DMEM, Gibco, Thermo Fisher Scientific) containing 10% fetal calf serum (FCS), 100 U penicillin and 100 mg streptomycin per mL at 37°C and 5% CO_2_.

### Plasmids

The pCAGGS expression plasmids encoding any of the H18N11 NP variants were generated by site directed mutagenesis via two-step assembly PCR using the Phusion polymerase (Thermo Fisher Scientific) and respective mismatch primer sets.

### Polymerase reconstitution assay

HEK293 T cells were seeded and grown in 12-well plates and subsequently transfected with pCAGGs expression plasmids encoding the polymerase subunits PB2, PB1 and PA (each 10 ng) of H5N1 together with 100 ng of pCAGGs plasmids coding for either H5N1-NP, H18N11-NP, H18N11-NP_L48Q_, H18N11-NP_A53D_, H18N11-NP_R98K_, H18N11-NP_K99R_, H18N11-NP_E100V_ or pH1N1-NP. The firefly luciferase-encoding construct pPolI-FFLuc-RT (50 ng) served as a viral minigenome. Transfection efficiency was determined by co-transfecting 10 ng of the pRL-SV40 plasmid coding for the *Renilla* luciferase. Additionally, 300 ng of pCAGGs plasmids expressing human MxA or C. perspicillata Mx1 (bMx1) or the respective inactive constructs MxA_T103A_ or Mx1_T103A_ (bMx1_T103A_) were transfected. At 24 h post transfection cells were lysed and the firefly and *Renilla* luciferase activities were measured by using the Dual-Luciferase^®^ reporter assay system (Promega).

### Viruses

Recombinant viruses A/Puerto Rico/8/1934 (PR8), A/Hamburg/4/2009 (pH1N1), A/Thailand/1(KAN-1)/2004 (H5N1) and the chimeric bat virus PR8-H18N11 were generated utilizing the eight-plasmid pHW2000-based rescue system [17]. All recombinant viruses were plaque purified and then used for stock generation. Stock titres were determined by plaque assay on MDCKII cells.

### Virus infections

MDCKII, MDCK-SIAT1, MDCK-SIAT1-MxA and MDCK-SIAT1-MxA_T103A_ cells were seeded and grown in 6-well plates. Prior to infection cells were washed with PBS (0.2% BSA) and then infected with virus at an MOI of 0.001 in infection medium (DMEM, containing 0.2% BSA and 100 U penicillin and 100 mg streptomycin per mL). For PR8, pH1N1 and PR8-H18N11 1 µg per mL TPCK-treated trypsin was added into the infection medium. Viral titres were determined by plaque assay.

### Western blot

Protein samples from cell lysates were incubated at 95°C in Laemmli buffer and subsequently separated by sodium dodecyl sulphate polyacrylamide gel electrophoresis (SDS–PAGE). Separated protein samples were blotted on a nitrocellulose membrane. MxA/b Mx1, NP or actin levels were determined using specific antibodies against the highly conserved G domain in Mx proteins (M143) [18], Flag (Sigma-Aldrich, F3165, 1:1000), NP (Gene Tex, GTX125989, 1:1000) and actin (Sigma-Aldrich, A3853; 1:1000), respectively. Primary antibodies were detected using Peroxidase-conjugated secondary antibodies (Jackson ImmunoResearch, 1:5000).

### Mice infections

All mouse experiments were performed in accordance with the guidelines of the German animal protection law and were approved by the state of Baden-Württemberg (Regierungspräsidium Freiburg; reference number: 35-9185.81/G-17/14). C57BL/6 mice (B6) lacking functional endogenous *Mx1* genes were obtained from Janvier, B6 A2G-Mx1 mice (Mx1) carrying the functional *Mx1* allele and human MxA transgenic mice (hMxA-tg) were bred locally and treated in accordance with guidelines of the Federation for Laboratory Animal Science Associations and the national animal welfare body. For survival analysis, 6–10 weeks old mice were anaesthetized with a mixture of ketamine (100 mg per g body weight) and xylazine (5 mg per g body weight) administered intraperitoneally and were subsequently inoculated intranasally with 40 µL of the indicated virus dose diluted in Opti-MEM containing 0.3% BSA. Changes in body weight were monitored daily and mice were sacrificed when 75% of the initial body weight was reached. For interferon pretreatment 2 μg per 100 μL IFN-α was administered subcutaneously 18 h prior to challenge with the indicated virus. At 1 and 3 days post infection mice were sacrificed and the upper respiratory tract, trachea and lung were dissected. Organs were homogenized in 1 mL PBS by three subsequent rounds of mechanical treatment for 25 s each at 6.5 ms^−1^. Tissue debris was removed by centrifuging homogenates for 5 min at 5000 rpm at 4°C and samples were stored at −80°C until further processing. Viral organ titres were determined by plaque assay.

### LD_50_ determination

The 50% median lethal dose was determined as described previously using at least four animals for each virus dilution and the method of Reed and Muench for calculation [19].

### Molecular modelling

The three-dimensional structure of H18N11 NP was generated by using I-Tasser (https://zhanglab.ccmb.med.umich.edu/I-TASSER/) and displayed using the PyMOL software (https://pymol.org/2/).

### Sequence alignment

Unless otherwise stated, NP sequences of PR8 (GenBank: AAA43467.1), pH1N1 (GenBank: HQ111365.1), Belzig [13], KAN-1 (GenBank: AFF60788.1) and H18N11 (GenBank: CY125946.1) were retrieved from GenBank and aligned with MEGA7.

## Results

### H18N11 NP confers partial MxA resistance in a polymerase reconstitution assay

**Figure 1.**
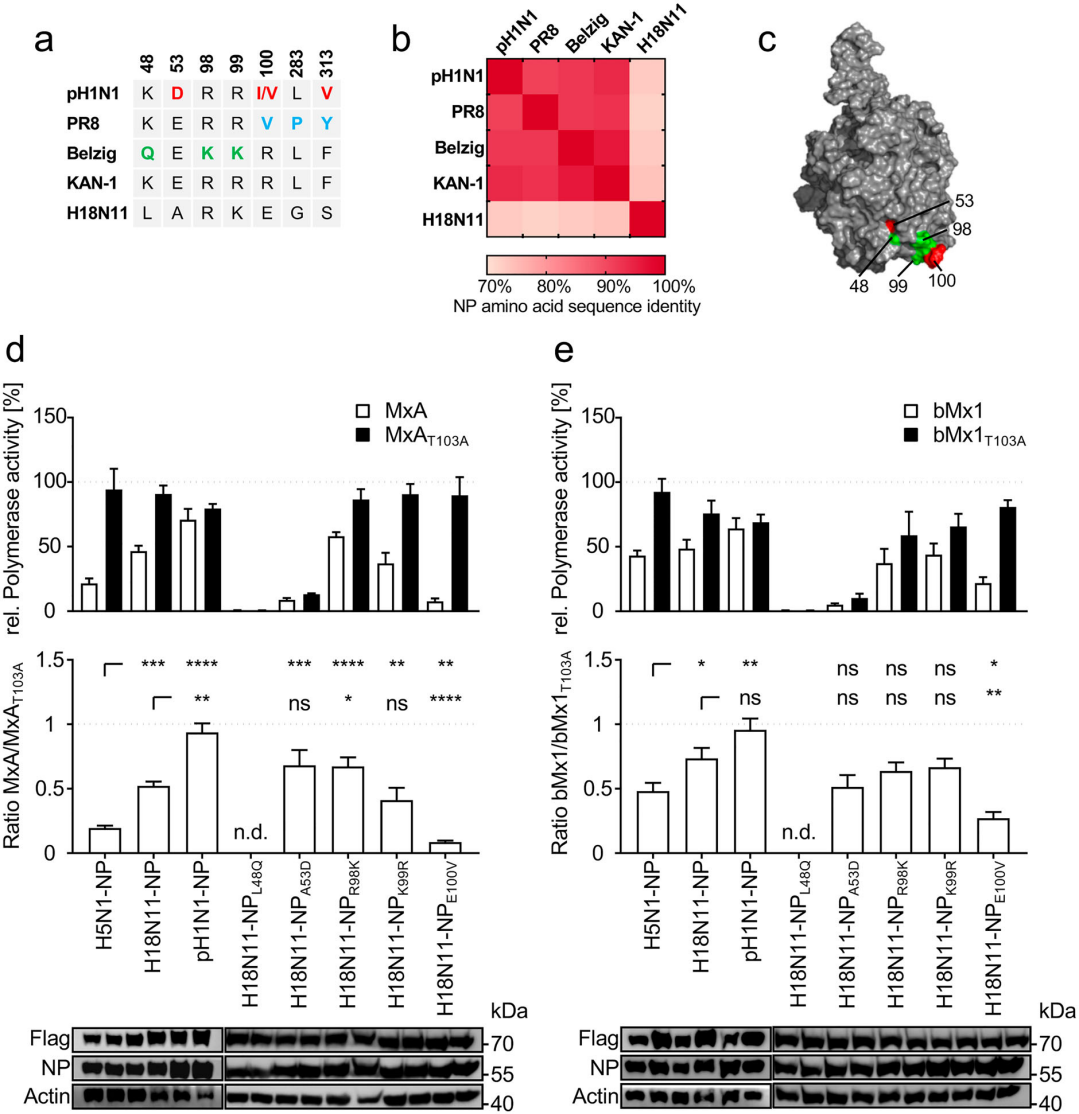
The bat-derived H18N11 NP confers intermediate MxA-resistance to the avian KAN-1 polymerase in polymerase reconstitution assay. (a) Amino acid differences in NP among indicated IAV strains. Previously described MxA escape mutations encoded by the human influenza strains pH1N1 (red) and PR8 (blue) and the Eurasian avian-like swine isolate Belzig (green) are highlighted. KAN-1 and H18N11 exhibit none of the known MxA-resistance patches. (b) NP amino acid sequences of the indicated strains were aligned to each other to determine amino acid sequence identity. (c) Known MxA escape mutations from (a) are highlighted in the modelled protein structure of the H18N11 NP. Adaptive mutations described for pH1N1 are shown in red and those for Belzig are displayed in green. Note that the amino acid positions 283 and 313 are not surface exposed. (d) HEK293 T cells were transfected with expression plasmids encoding the avian KAN-1 polymerase subunits PB2, PB1 and PA together with the indicated NP variants. At 24 h post transfection the viral polymerase activity was determined in presence of human MxA (white bars) or the inactive MxA_T103A_ (black bars) and normalized to the empty vector control that was set to 100% (upper panel). The ratios of MxA to MxA_T103A_ activities are indicated in the lower panel. (e) Viral polymerases with the indicated NP variants were reconstituted as described in (d) and expression plasmids encoding *C. perspicillata* bMx1 (white bars) or the inactive bMx1_T103A_ (black bars) were co-transfected and normalized to the empty vector control (upper panel). The ratios of bMx1 to bMx1_T103A_ activities are indicated in the lower panel. Expression levels of Flag tagged MxA or bMx1 and NP were detected by Western blot. Error bars indicate the standard error of the mean of at least three independent experiments. n.d., not done; ns, not significant; ** *P* < 0.01; *** *P* < 0.001; **** *P* < 0.0001; Student’s *t* test.

It was previously reported that NP determines the Mx sensitivity of IAVs within the respective hosts [8,13,20]. While the human-adapted IAV strains PR8 and A/Hamburg/4/2009 (pH1N1) as well as the Eurasian avian-like swine isolate A/swine/Belzig/2/2001 (Belzig) harbour specific and partially overlapping clusters of MxA-escape mutations in NP [8,13], these mutations are virtually absent in avian IAVs of the H5N1 subtype such as A/Thailand/1(KAN-1)/2004 (KAN-1) (Figure 1(a)). Similarly, the bat influenza-derived H18N11 NP lacks most known MxA-resistance mutations, with exception of K99 that is also found in Belzig NP (Figure 1(a)). Moreover, while NPs of conventional IAVs share a high degree of amino acid identity among each other (91% to 96%), the H18N11 NP exhibits only less than 75% identity to conventional IAV NPs (Figure 1(b)). Thus, we wondered whether H18N11 NP harbours a novel cluster of MxA escape mutations, including K99 (Figure 1(c)) or, alternatively, lacks sufficient adaptive mutations to overcome MxA restriction. To test this, we determined the MxA sensitivity of H18N11 NP in a polymerase reconstitution assay, using the avian KAN-1 polymerase together with either KAN-1, H18N11 or pH1N1 NP and co-transfected MxA or the inactive variant MxA_T103A_. By determining the ratio of the relative polymerase activity in presence of MxA or MxA_T103A_, we estimated the level of MxA resistance conferred by the respective NP. As expected, the viral polymerase reconstituted with the avian KAN-1 NP was highly sensitive to MxA, whereas NP from pH1N1 rendered the viral polymerase MxA-resistant (Figure 1(d)). Interestingly, the H18N11 NP revealed an intermediate phenotype between KAN-1 and pH1N1 NP, suggesting that the H18N11 NP is able to mediate partial MxA resistance (Figure 1(d)). By utilizing site-directed mutagenesis we introduced then known MxA-escape amino acids shown in Figure 1(a) into H18N11 NP and tested their effect on MxA resistance. We observed that both amino acid substitutions NP_L48Q_ and NP_A53D_ greatly impaired the polymerase activity. In contrast, NP_E100V_ did not affect the polymerase activity but considerably decreased the MxA resistance, whereas substitution of R98 with a lysine (NP_R98K_) increased MxA resistance compared to H18N11 NP. However, substituting the potential MxA escape mutation in H18N11 NP at K99 (Figure 1(a)) with an arginine (NP_K99R_) did not result in increased MxA sensitivity. Next we analysed the antiviral function of the bMx1 protein from *C. perspicillata* against the different IAV NPs. Although to a lesser extent than MxA, *C. perspicillata* bMx1 restricted the viral polymerase activity in the context of KAN-1 NP, but had no or only little effect on pH1N1 and H18N11 NP, respectively (Figure 1(e)). Moreover, in the context of bMx1, both amino acid substitutions at R98 (R98 K) and K99 (K99R) had no effect on the polymerase activity, whereas the mutation E100 V decreased bMx1 resistance. Based on these results we conclude that H18N11 NP not only confers partial Mx resistance to *C. perspicillata* bMx1 but also towards human MxA.

### MxA suppresses viral replication of PR8-H18N11 *in vitro*

**Figure 2.**
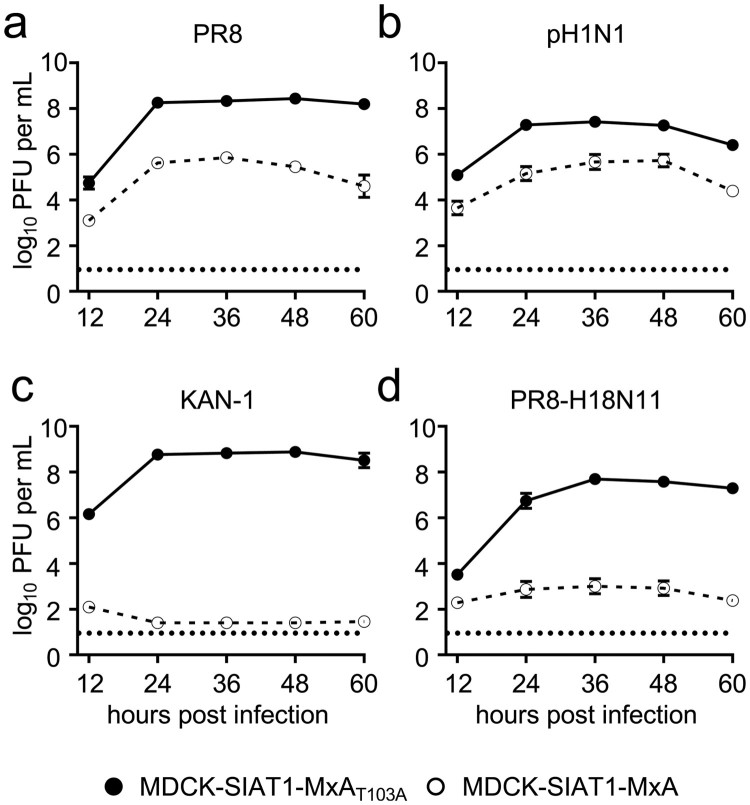
Viral growth of the chimeric bat influenza virus PR8-H18N11 is suppressed by MxA *in vitro*. MDCK-SIAT1 cells overexpressing MxA (dashed line) or MxA_T103A_ (solid line) were infected with (a) PR8, (b) pH1N1, (c) KAN-1 or (d) PR8-H18N11 at an MOI of 0.001 and viral titres were measured at 12, 24, 36, 48 and 60 h post infection by plaque assay. Error bars indicate the standard error of the mean of at least three independent experiments. Dashed line indicates the detection limit.

Next we aimed to evaluate whether MxA restricts viral growth of H18N11 in cell culture. For this, we generated a chimeric bat IAV encoding six out of eight gene segments from H18N11 together with two gene segments encoding the surface glycoproteins of the prototypic IAV strain PR8, designated PR8-H18N11 [21,22]. We further included the human IAV strains PR8 and pH1N1 as positive controls and the avian IAV KAN-1 as a negative control for MxA resistance in our analysis, and infected MDCK-SIAT1 cells stably overexpressing MxA (MDCK-SIAT1-MxA) or the inactive counterpart MxA_T103A_ (MDCK-SIAT1-MxA_T103A_) at an MOI of 0.001. Consistent with previous results [13], PR8 and pH1N1 demonstrated robust but decreased replication in the MxA-expressing cell line over time compared to MDCK-SIAT1-MxA_T103A_ cells (Figure 2(a,b)). As expected, KAN-1 replicated efficiently in MDCK-SIAT1-MxA_T103A_ cells, resulting in the release of >10^8^ infectious virus particles per mL, but viral growth was completely abolished upon MxA overexpression (Figure 2(c)). Intriguingly, replication of PR8-H18N11 was similarly restricted in MDCK-SIAT1-MxA cells as depicted by low viral titres with ∼10^3^ PFU per mL in the cell supernatant between 24–60 h post infection (Figure 2(d)). These results indicate that the bat IAV H18N11 is, similarly to the avian influenza virus KAN-1, highly sensitive to the antiviral activity of human MxA in MDCK-SIAT1 cells.

### hMxA-tg and Mx1 mice are partially resistant to infection with PR8-H18N11

**Figure 3.**
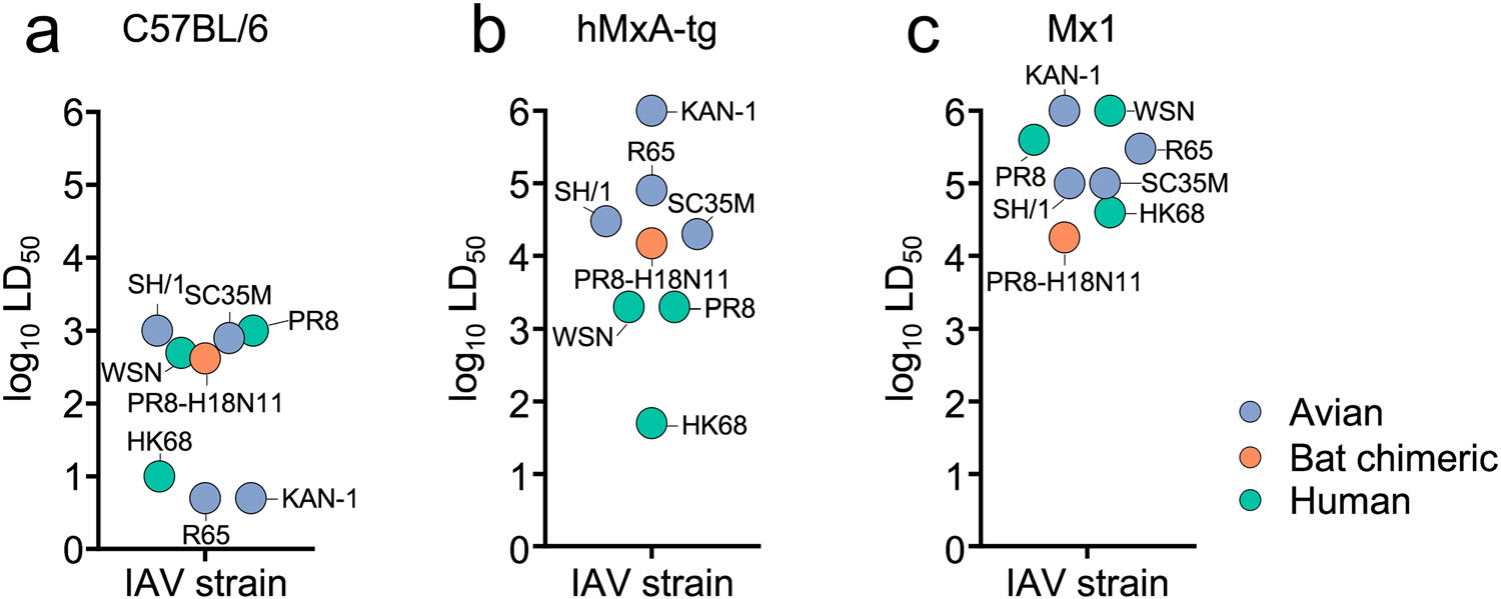
Replication of PR8-H18N11 is controlled by MxA and Mx1 *in vivo*. Groups of (a) B6, (b) hMxA-tg and (c) Mx1-positive mice were challenged with chimeric PR8-H18N11 to determine LD_50_ values. Obtained LD_50_ values were merged with the previously determined LD_50_ values of avian (KAN-1, R65, SH/1 and SC35M) and human IAVs (PR8, WSN and HK68) in B6, hMxA-tg and Mx1-positive mice [12].

To provide proof for the importance of MxA in controlling viral growth of PR8-H18N11 *in vivo*, we determined the median lethal dose (LD_50_) of this chimeric bat virus in the recently generated transgenic C57BL/6 (B6) mice encoding the human *MX1* gene locus (hMxA-tg) [12] in comparison to control B6 mice lacking a functional murine Mx1. Thereby, it can be assessed to which extent IAV strains are adapted to overcome the antiviral activity of MxA. Consistent with a high degree of MxA resistance, it has been previously shown that the LD_50_ values of IAVs of human origin, such as PR8, A/WSN (H1N1) and A/HK68 (H3N2) are only slightly increased (2-to 5-fold) in hMxA-tg mice relative to control B6 mice (Figure 3(a,b)) [12]. B6 mice encoding a functional murine *Mx1* gene locus (Mx1) are known to be highly resistant to infections with influenza viruses of both human and avian origin such as KAN-1, A/R65 (H5N1), A/SH/1 (H7N9) and A/SC35M (H7N7) (Figure 3(c)) [12]. Intriguingly, we found that the LD_50_ value of the chimeric PR8-H18N11 virus was 35-fold higher in hMxA-tg mice (LD_50_ = 1.5 × 10^4^ PFU) relative to B6 mice (LD_50_ = 4.2 × 10^2^ PFU) (Figure 3(a,b)). Surprisingly, Mx1-positive mice demonstrated a comparable resistance as their transgenic MxA counterparts towards PR8-H18N11 challenge (LD_50_ = 1.8 × 10^4^ PFU) (Figure 3(b,c)).

**Figure 4.**
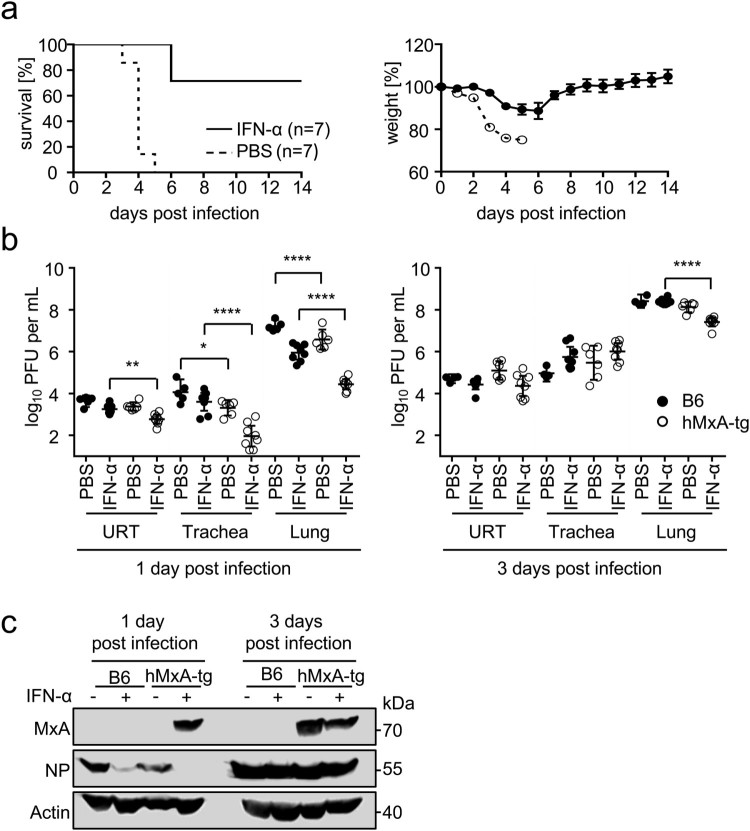
hMxA-tg mice resist lethal dose of PR8-H18N11. (a) hMxA-tg mice were pretreated with IFN-α or PBS 18 h prior to infection with 7xLD_50_ of PR8-H18N11 (values refer to hMxA-tg mice). Survival and changes in body weight were monitored for 14 days. Mice were sacrificed and scored dead when 75% of the initial body weight was reached. Error bars indicate the standard error of the mean. (b) Groups of B6 and hMxA-tg mice were pretreated with IFN-α or PBS 18 h prior to infection with 7xLD_50_ of PR8-H18N11 (values refer to hMxA-tg mice). Viral organ titres of the upper respiratory tract (URT), trachea and lung were determined at the indicated time points. (c) MxA and NP protein levels in homogenized lungs from infected mice from (b) were detected by Western blot.

To demonstrate that MxA protects against lethal doses of PR8-H18N11 in hMxA-tg mice under more stringent conditions, we induced MxA by treating these mice with IFN-α 18 h prior to challenge with 7x LD_50_ of the chimeric bat virus. Under these conditions PR8-H18N11 caused only slight weight loss, and most transgenic mice survived the lethal infection dose, whereas all unstimulated control animals were unable to withstand the lethal infection (Figure 4(a)). IFN-α pretreatment of hMxA-tg mice reduced viral titres ∼3-fold in the upper respiratory tract, ∼44-fold in the trachea and ∼33-fold in the lung compared to IFN-α stimulated B6 control mice at 1 day post infection. At 3 days post infection we determined ∼9-fold differences in viral lung titres between IFN-α-treated transgenic mice and B6 mice (Figure 4(b)), highlighting the role of MxA in restricting viral growth. Consistently, Western blot analysis of lung homogenates demonstrated clear MxA induction and the absence of detectable NP levels in IFN-α-treated hMxA-tg mice at 1 day post infection. Sanger sequencing of isolated viral RNA from hMxA-tg lung homogenates taken at 3 days post infection confirmed the absence of mutations in NP. Overall, these data demonstrate that MxA is a potent restriction factor that protects against lethal infection doses of PR8-H18N11.

## Discussion

The interferon-induced human MxA protein represents a major interspecies barrier for a broad variety of zoonotic viruses, including IAV [23]. To overcome this innate immune restriction, IAVs have to acquire MxA escape mutations in NP, which are invariably found in all known human-adapted IAV strains [8,11]. It is now becoming evident that such MxA escape mutations can be acquired in intermediate hosts such as swine, which express an antivirally active Mx1 protein [8,13]. Intriguingly, the newly discovered bat-derived influenza A-like virus H18N11 circulates in bat species that encode antivirally active bMx1 proteins, raising the potential of its MxA preadaptation. Here we show that NP of H18N11 confers only moderate MxA resistance in a polymerase reconstitution assay. Furthermore, viral growth of the chimeric bat PR8-H18N11 virus was severely attenuated upon MxA overexpression *in vitro* and also in transgenic mice encoding human *MxA*, indicating that H18N11 NP is not sufficiently preadapted to facilitate MxA escape. Nevertheless, the residual MxA resistance of H18N11 NP in the polymerase reconstitution assay suggests that a certain degree of Mx preadaptation was already obtained by the selection pressure functional bMx1 proteins. Especially E100 in the H18N11 NP, but not the Belzig resistance determinant K99, appeared to be important for partial MxA and bMx1 escape. Surprisingly, substitution of E100 in H18N11 NP with a known MxA escape mutation (E100 V) considerably decreased Mx resistance, suggesting a limited compatibility with known MxA escape mutations found in NP of human IAVs. However, since only few H18N11 genomic sequences are known to date [15,24], it remains unclear whether other H18N11 variants exist that encode additional Mx escape mutations in NP that also increase MxA resistance.

Human influenza virus strains are able to overcome MxA restriction but are efficiently controlled in mice expressing endogenous *Mx1* genes [12], an observation that is attributed to a stronger antiviral activity of mouse Mx1 compared to human MxA. Surprisingly, however, both hMxA-tg and Mx1 positive mice were similarly resistant to challenges with the chimeric bat virus PR8-H18N11, resulting in comparable LD_50_ values that were ∼36-fold and ∼43-fold higher compared to B6 control mice (Figure 3(a–c)). This might reflect strain-specific differences in the replication efficiency of human IAVs and PR8-H18N11 in mice that are possibly associated with differences in the induction of the innate immune response or its suppression. Hence, true Mx resistance might be only unveiled upon IFN stimulation prior to infection. Under these specific conditions, even lethal doses of PR8-H18N11 barely caused weight loss and mortality (Figure 4(a)), demonstrating the MxA sensitivity of the bat virus.

In summary, our data suggest that the IFN-induced human MxA protein efficiently controls the novel bat influenza A-like virus H18N11, thereby significantly reducing its zoonotic risk. However, due to a weak but detectable antiviral activity of *C. perspicillata* Mx1 it is difficult to predict whether sufficient MxA-escape mutations in H18N11 NP can be acquired that ultimately result in full MxA resistance.
